# Comparative Analysis of Edentulism in a Sample of Mexican Adults with and without Type 2 Diabetes

**DOI:** 10.3390/healthcare10122378

**Published:** 2022-11-26

**Authors:** Rosalina Islas-Zarazúa, Mariana Mora-Acosta, José de Jesús Navarrete-Hernández, Josefina Reynoso-Vázquez, Juan José Villalobos-Rodelo, Laura Rojas-Ortega, Taurino Amilcar Sosa-Velazco, María de Lourdes Márquez-Corona, Carlo Eduardo Medina-Solís, Gerardo Maupomé

**Affiliations:** 1 Dentistry Academic Area, Health Sciences Institute, Autonomous University of the State of Hidalgo, Pachuca 42160, Mexico; 2 Pharmacy Academic Area, Health Sciences Institute, Autonomous University of the State of Hidalgo, Pachuca 42160, Mexico; 3 School of Dentistry, Autonomous University of Sinaloa, Culiacan 80040, Mexico; 4 Department of Epidemiology, Institute of Social Security and Services for Government Workers, Culiacan 80000, Mexico; 5 Universidad Contemporánea de las Américas, Ciudad de Mexico 04890, Mexico; 6 School of Dentistry, Autonomous University “Benito Juarez” of Oaxaca, Oaxaca de Juárez 68120, Mexico; 7 Advanced Studies and Research Center in Dentistry “Dr. Keisaburo Miyata”, School of Dentistry, Autonomous University of State of Mexico, Toluca 50000, Mexico; 8 Richard M. Fairbanks School of Public Health, Indiana University/Purdue University, Indianapolis, IN 46202, USA; 9 Indiana University Network Science Institute, Bloomington, IN 47408, USA

**Keywords:** edentulism, diabetes, prevalence, epidemiology, adults, dental setting, Mexico

## Abstract

The objective of the present study was to compare the prevalence of edentulism in Mexican adults with and without a diagnosis of type 2 diabetes mellitus (T2DM) when they are seeking dental care. A cross-sectional study was conducted on 1921 medical records of Mexican adults 40 years of age and older who sought dental care at clinics of a public university in Mexico. The dependent variable was edentulism, clinically determined through an oral examination. The main independent variable was the self-report of previous T2DM diagnosis made by a physician. Sociodemographic, socioeconomic and behavioral covariates were included in a multivariate binary logistic regression model. Overall edentulism prevalence was 8.4% (95% CI = 7.1–9.6). The prevalence of T2DM was 14.3% (n = 274). The prevalence of edentulism among individuals with T2DM was 13.1%, but only 7.6% among individuals without T2DM. In the multivariate binary logistic regression model, a previous T2DM diagnosis increased the probability of being edentulous 1.61 times (95% CI = 1.03–2.50). For each year a person’s age increased, the likelihood of being edentulous increased by 12% (95% CI = 10–14%). In summary, a higher prevalence of edentulism was present in Mexican adults with T2DM and in those of older age. This information may be used by dental care providers and health policymakers to improve approaches to preventive care, as well as to characterize and anticipate care needs more accurately for the adult and older adult populations.

## 1. Introduction

Due to the high prevalence and incidence of oral diseases, the total number of individuals they affect, and their economic impact on families and on health systems, they are considered public health problems [1,2]. Epidemiological studies have consistently shown that poor oral health is a major health challenge around the world; it has been poorly addressed in general. Globally, there were 3.5 billion cases of oral conditions: 2.3 billion had untreated caries in permanent teeth, 796 million had severe periodontitis, 532 million had untreated caries in primary teeth, 267 million had complete tooth loss, and 139 million had other oral conditions in 2017 [3]. There is conflicting evidence about trends and mechanisms leading to increasing and decreasing edentulism rates. Edentulism is generally declining in developed countries, but generally increasing in developing countries. Because of aging and the increasing number of older adults reaching old age, edentulism continues to grow [4]. A recent meta-analysis focused on 45-year-olds and older reported a range of prevalence figures between 1.1% and 70%, with a combined 22.0% prevalence worldwide [5]. According to a national survey in Mexico, approximately 10% of adults between 45 and 54 years old, 25% between 65 and 74 years old, and 30% 65 and older, are edentulous [6]. Documenting trends in tooth loss may help in planning dental care services and workforce needs [7]. Edentulism connotes a disability condition that reflects a personal history of severe oral disease and access to dental services throughout life [7,8]; such history is primarily driven by caries and periodontitis [9,10,11]. Tooth loss and edentulism negatively impact people’s nutritional intake. People with more severe tooth loss may consume significantly fewer basic nutrients from fruits and vegetables, dietary fiber, and protein, compared to those without severe tooth loss [8,12,13,14]. Evidence from observational studies showed that tooth loss and edentulism may be associated with multiple adverse health effects [15].

The most prevalent chronic diseases share some modifiable risk factors with oral diseases and, therefore, may occur in the same patients [2,16,17,18,19]. In addition, people with chronic diseases are more likely to have untreated dental disease and periodontitis, which may lead to tooth loss [2,20,21,22,23,24]. Oral diseases and diabetes mellitus are an example of such overlap [25,26,27,28]. There are three types of diabetes: type 1, type 2, and gestational [29]. T1DM may occur at any age but tends to strike earlier in life. T2DM is more common in adults and accounts for 90% of all diabetes cases. T2DM is characterized by elevated blood glucose resulting from insufficient insulin production, inadequate effect of existing insulin, or both [29,30]. Mexico is a country with some of the highest diabetes prevalence rates in the world [31]; survey data have shown their continuous increase [32]. It is estimated that by 2030 the prevalence will reach 12–18% and, by 2050, 14–22% [33]. Under such a scenario, it would be reasonable to expect that edentulism would increase accordingly; that situation calls for greater attention by the health care and the health promotion systems to address potential additional impacts.

The bidirectional link between diabetes and periodontitis has been well established: through various mechanisms, diabetes promotes the destruction of periodontal tissues and periodontal disease negatively affects glycemic control [34]. The prevalence of edentulism in patients with T2DM is associated with oral problems such as salivary gland hypofunction, periodontitis, root caries, and pulpal involvement after severe carious lesions, as well as being associated with general health factors such as depression, cognitive impairment, and pain [35,36]. With T2DM increasing worldwide, it is reasonable to expect an increase in dental care needs [36]. Because the association between edentulism and T2DM has been reported to hold diverse directions and/or strengths [2,37,38], it is plausible that such association is context-specific [35]. We sought to expand the knowledge base. The aim of the present study was to compare the prevalence of edentulism in Mexican adults with and without a diagnosis of T2DM when they seek dental care. The hypothesis was that a group of people with T2DM would have a higher percentage of edentulism, compared to those without.

## 2. Materials and Methods

### 2.1. Study Design and Location

This is a secondary analysis of a cross-sectional study in a random sample of medical records of adult patients. They were seeking dental care at the clinics of the Dentistry Academic Area of the Autonomous University of the State of Hidalgo. The original sample was of individuals over 17 years of age; but for the present sub-analysis, we only included those 40 years of age and older. The methodology has been previously published [39,40]. For the calculation of the sample size in the original study, the following parameters were used: the universe was 16,500 medical records, the heterogeneity (diversity) was 50%, the margin of error was 2%, a confidence level of 99%, and a 5% loss, so a sample of 3481 medical records was obtained. For the present analysis, the inclusion criteria were medical records of individuals of: (1) any sex, (2) 40 years of age or older, (3) subjects who were seeking dental care at the university clinics. The exclusion criteria were: (1) incomplete medical records, (2) medical records absent at the time of the study, (3) medical records of patients diagnosed with T1DM. After the inclusion and exclusion criteria were applied, the final sample for the analysis of the present study was 1921. The power in the present study was 0.80 to detect significant differences (alpha = 0.05) between this sample and the observed proportions.

### 2.2. Study Variables

The medical/dental records accruing data for the present study were collected by senior dental students. They were trained and standardized in the criteria used.

The dependent variable was edentulism, which was coded as 0 = patient without edentulism and 1 = patient with edentulism. The main independent variable was the self-report of having received a T2DM diagnosis by a physician, which was dichotomized as 0 = no, 1 = yes. Other covariates were: age (40 to 95 years old, as a continuous variable); sex (0 = female, 1 = male); type of housing (0 = good, 1 = fair, 2 = poor); marital status (0 = with partner, 1 = without partner); health insurance (0 = none, 1= any insurance, private or public); reason for dental care (0 = preventive, 1 = curative/rehabilitation); and high blood pressure (0 = no, 1 = yes). Socioeconomic position (SEP) (measured through occupation as: 0 = low SEP, 1 = medium, 2 = high, together with type of housing). Occupation was derived from the National Occupational Classification System and subsequently categorized into levels 0 = low, 1 = medium and 2 = high [41].

### 2.3. Statistical Analysis

A univariate analysis was performed: measures of central tendency and dispersion are reported for the continuous variables and frequencies and percentages for the categorical variables.

A binary logistic regression model was used for the bivariate and multivariate analyses. The strength of the association between the dependent variable (edentulism) and the independent variables was expressed as odds ratio (OR) with 95% confidence intervals (95% CI). The variance inflation factor (VIF) test was performed to analyze and, if necessary, avoid multicollinearity between the independent variables. For the construction of the model, those variables that in the bivariate analysis showed a value of *p* < 0.25 were taken into account. The overall fit of the model was performed using the Hosmer and Lemeshow goodness-of-fit test [42]. The Stata statistical software package (version 14) was used for all analyses (StataCorp LP; College Station, TX, USA).

### 2.4. Ethical Statement

The research and ethical guidelines of the Helsinki principles were followed to conduct this study. The study included identified medical/dental records whose patients had consented to the use of clinical information for research as long as data privacy was guaranteed. The project was approved by the ethics and research committee of the Institute of Health Sciences of the Autonomous University of the State of Hidalgo (CEEI-000037-2019).

## 3. Results

A total of 1921 individuals were included in the present study. Their characteristics are presented in Table 1. The mean age was 53.9 ± 10.8. Most were women (64.4%). Most had a partner (62.3%). Regarding socioeconomic factors, the majority (85.0%) were classified as having a “low” level occupation but with “good” housing characteristics. Almost all (93.2%) of the individuals had access to health insurance, either public or private; 69.8% sought dental curative treatment or rehabilitation at the clinic, and almost 60% had a diagnosis of high blood pressure. A previous diagnosis of T2DM was found in 14.3% (95% CI = 12.7–15.8) of records. The prevalence of edentulism was 8.4% (95% CI = 7.1–9.6).

Table 2 presents the results of the bivariate analyses. The prevalence of edentulism among individuals with T2DM was 13.1% and 7.6% among individuals without (OR = 1.84, 95% CI = 1.10–1.14, *p* = 0.002). The mean age was higher among individuals with edentulism (68.3 ± 10.8 years) compared with individuals without edentulism (52.6 ± 9.8): (OR = 1.12, 95% CI = 1.10–1.14). Individuals without a partner were 1.66 (95% CI = 1.20–2.29) times as likely to be edentulous than individuals with a partner.

The multivariate binary logistic regression model is presented in Table 3; it was observed that a previous diagnosis of T2DM increased the chance of being edentulous by 61% (95% CI = 1.03–2.50). For each year’s increase in age, the likelihood of being edentulous increased by 12% (95% CI = 10–14%).

## 4. Discussion

The aim of the present study was to compare the prevalence of complete tooth loss between two groups of Mexican adults, one with a diagnosis of T2DM and the other without such a diagnosis. The results showed that in the group with T2DM the prevalence of edentulism was higher than in the group without T2DM. The overall prevalence of edentulism was 8.4%, resembling results in populations of similar age. In a previous study conducted in 20 states of Mexico (out of 32 total) [37], a 10.2% prevalence of edentulism was observed in individuals ages 35 years and older, with variations among states ranging from 5.0% to 16.7%. Although the prevalence of edentulism has decreased in several countries, in Mexico it is estimated that 6.3% (N = 3,437,816) of the population ages 18 years and older are edentulous [6]. In a multi-country study, the overall prevalence of edentulism was 11.7% [38], with India, Mexico, and Russia having the highest prevalence (16.3% to 21.7%), and with China, Ghana, and South Africa (3.0% to 9.0%) having the lowest. In individuals older than 30 years in Iran, prevalence between 3.0% and 78.0% has been reported [43]. These wide ranges can be explained by multiple factors, such as the characteristics of each country studied, the distribution of socioeconomic groups within these countries, the response of the health systems to the oral health needs of the population, the actual access to relevant health services, the age groups included, or the methodology used to collect data (clinical or self-reported). Direct comparisons between our results and those of other studies may only allow partial conclusions to be drawn. However, it is important to emphasize that quantifying edentulism prevalence in adults is a valuable indicator of treatment needs—felt, normative, and met. These evaluations help estimate the proportion of the population that is expected to use dental services in the future, thus providing essential information for dental service planning and human resource training.

Oral diseases such as caries [44,45,46,47,48] and periodontal diseases [49,50,51,52,53] are considered public health problems in Mexico due to their high prevalence and incidence, as well as the ensuing high treatment needs. Mexico is one of the countries with the highest prevalence and incidence of T2DM [31,32]. This combination does not foretell the best outcomes for oral health, and it is likely/will likely be associated with a high edentulism experience [2,20,21,24]. In the present study, T2DM was a risk factor for edentulism as reported in other studies [8,9,21,34,36]. In recent years, the connection between oral health and systemic health has been widely recognized by the dental and medical professions. Studies have been conducted revealing that oral health affects systemic health and vice versa, subject to the influence of inflammatory mechanisms [8], with molecular and immunological bases [22]. These diseases/conditions share common risk factors, so comprehensive promotion of general health and oral health focused on addressing unhealthy behaviors has the potential to reduce both dental disease rates, as well as mortality rates attributed to cardiovascular disease, cancer, and T2DM [19]. Both families and the health care system in Mexico may be negatively affected because of the high cost of addressing T2DM and edentulism.

The present study confirmed that older age was associated with edentulism, as previously reported [8,20,21,36]. Tooth loss is considered an accurate marker of the population’s oral health and is therefore monitored in many countries. Edentulism reflects not only dental disease but also the attitudes of patients and dentists, the dentist-patient relationship, the availability and accessibility of dental services, and the prevailing philosophies of dental care [7]. Self-care, as behaviors that each individual must deliberately apply in order to maintain good health, may be an ideal component of the approach to ameliorate the impact of risk factors (modifiable and non-modifiable) [54].

The present study has certain limitations. The first is the cross-sectional design and its temporal ambiguity; by measuring cause and effect at the same time, causal relationships beyond statistical associations cannot be established. Another possible source of bias may be the self-reported data of T2DM, although self-reported health data are known to be valid and reliable in many cases; there is no obvious reason for patients to conceal their T2DM when reporting medical history features. We were circumscribed to analyzing data collected from medical/dental histories derived from a real-life dental education environment; although procedures undertaken by senior students were highly standardized and scrutinized, data quality is of necessity not as good as in research studies.

## 5. Conclusions

In this sample of Mexican adults ages 40 years and older, edentulism prevalence was 8.4%. A higher prevalence was observed among individuals with T2DM. Age was also associated with edentulism. This information is useful for dental care providers and health policymakers to improve approaches to preventive care, as well as to be able to characterize more accurately the care needs of the adult and older adult population.

## Figures and Tables

**Table 1:** Descriptive analysis of the study sample of Mexican adults aged 40 years and older.

Variables	Mean ± SD
Age	53.91 ± 10.84
	n (%)
T2DM No Yes	1647 (85.7) 274 (14.3)
Sex Female Male	1237 (64.4) 684 (35.6)
Marital status With partner Without partner	1196 (62.3) 725 (37.7)
Occupation Low Medium High	1633 (85.0%) 159 (8.3) 129 (6.7)
Housing Poor Fair Good	30 (1.6) 662 (35.5) 1229 (63.9)
Health insurance None Some insurance	130 (6.8) 1791 (93.2)
Reason for consultation Preventive Curative/rehabilitation	580 (30.2) 1341 (69.8)
High blood pressure No Yes	782 (40.7) 1139 (59.3)

SD: Standard Deviation.

**Table 2:** Bivariate analyses of the prevalence of edentulism across the independent variables included in the study.

Variables	Without Edentulism	With Edentulism	OR (95% CI)	*p*-Value
T2DM No Yes	1522 (92.4) 238 (86.9)	125 (7.6) 36 (13.1)	1 * 1.84 (1.24–2.73)	0.002
Age	52.59 ± 9.84	68.33 ± 10.85	1.12 (1.10–1.14)	<0.001
Sex Female Male	1140 (92.2) 620 (90.6)	97 (7.8) 64 (9.4)	1 * 1.21 (0.87–1.68)	0.252
Marital status With partner Without partner	1114 (93.1) 646 (89.1)	82 (6.9) 79 (10.9)	1 * 1.66 (1.20–2.29)	0.002
Occupation Low Medium High	1504 (92.1) 137 (86.2) 119 (92.2)	129 (7.9) 22 (13.8) 10 (7.8)	1.02 (0.52–1.99) 1.91 (0.87–4.19) 1 *	0.952 0.107
Housing Poor Fair Good	26 (86.7) 611 (92.3) 1123 (91.4)	4 (13.3) 51 (7.7) 106 (8.6)	1.62 (0.55–4.75) 0.88 (0.62–1.52) 1 *	0.371 0.489
Health insurance None Some insurance	122 (93.8) 1638 (91.5)	8 (6.2) 153 (8.5)	1 * 1.42 (0.68–2.96)	0.345
Reason for consultation Preventive Curative/rehabilitation	541 (93.3) 1219 (90.9)	39 (6.7) 122 (9.1)	1 * 1.38 (0.95–2.01)	0.085
High Blood Pressure No Yes	719 (91.9) 1041 (91.4)	63 (8.1) 98 (8.6)	1 * 1.07 (0.77–1.49)	0.670

* Reference category.

**Table 3:** Multivariate logistic regression model between edentulism and the independent variables.

Variable	OR (95% CI)	*p*-Value
Age	1.12 (1.10−1.14)	<0.001
T2DM No Yes	1 * 1.61 (1.03−2.50)	0.034

Hosmer-Lemeshow chi^2^ (8) = 13.06, *p* = 0.1097. Note: Estimates adjusted for the variables contained in the table, in addition to sex, marital status, and reason for consultation.

## Data Availability

The datasets generated during and/or analyzed during the current study are available from the corresponding author upon reasonable request.
